# Pulmonary embolism is more prevalent than deep vein thrombosis in cases of chronic obstructive pulmonary disease and interstitial lung diseases

**DOI:** 10.1186/s40064-016-3475-8

**Published:** 2016-10-12

**Authors:** Sun Hyo Park

**Affiliations:** Department of Internal Medicine, Keimyung University Dongsan Medical Center, 56 Dalseong-ro, Jung-gu, Daegu, 41931 Republic of Korea

**Keywords:** Chronic obstructive pulmonary disease, Interstitial lung disease, Pulmonary embolism, Deep vein thrombosis, Prevalence

## Abstract

**Background:**

Chronic lung diseases may have an influence on pulmonary vessel walls as well as on pulmonary haemodynamics. However, there is limited data on the occurrence of pulmonary embolism (PE) and deep vein thrombosis (DVT) in patients with chronic lung diseases, which have the potential to contribute to the development of pulmonary vascular abnormalities. We aimed to explore the prevalence of PE and DVT in patients with COPD and ILD.

**Methods:**

We evaluated the venous thromboembolism prevalence associated with COPD and ILD using Korean Health Insurance Review and Assessment Service (HIRA) data from January 2011 to December 2011. This database (HIRA-NPS-2011-0001) was created using random sampling of outpatients; 1,375,842 sample cases were collected, and 670,258 (age ≥40) cases were evaluated. Patients with COPD, ILDs, or CTD were identified using the International Classification of Disease-10 diagnostic codes.

**Results:**

The PE prevalence rates per 100,000 persons for the study population with COPD, ILD, CTD, and the general population were 1185, 1746, 412, and 113, respectively, while the DVT prevalence for each group was 637, 582, 563, and 138, respectively.

**Conclusions:**

PE prevalence was significantly higher than that of DVT in patients with COPD or ILDs, while the prevalence of PE was lower than that for DVT in the general population or in patients with CTD.

## Background

The pathogenesis of pulmonary embolism (PE) explained by deep venous thrombosis (DVT), develops in the lower extremities where clots may dislodge and travel through the inferior vena cava eventually lodging in the pulmonary arteries (Elliott 1992). Small clots from the lower extremities may start to lyse immediately and may resolve without serious clinical deterioration.

According to Virchow, venous thrombosis can be explained by blood stagnation (stasis), hypercoagulability, and vascular endothelial damage (endothelial dysfunction) (Bagot and Arya 2008). Venous blood flow generation is partly contributed to by the contraction of the muscle. It is thought that the pulmonary artery is unlikely to experience vascular injury from external factors. However, chronic lung diseases such as chronic obstructive pulmonary disease (COPD) or interstitial lung disease (ILD) may have an effect on the pulmonary circulation. In addition to airway and lung parenchymal abnormalities, COPD is also characterized by pulmonary vascular alterations, including medial wall thickening and muscularization of nonmuscularized arterioles (Magee et al. 1988; Wright et al. 1992). There have been studies that have focused on the vascular abnormalities experienced in ILDs, such as increased angiogenic chemokines, aberrant angiogenesis, and capillary endothelial abnormalities (Keane et al. 1997; Cosgrove et al. 2004; Renzoni et al. 2003; Sakao et al. 2006).

Finally, the higher prevalence of pulmonary hypertension in patients with ILDs and COPD may contribute to increased vascular resistance of the pulmonary vascular beds as well as stagnation of blood flow (Chaouat et al. 2008; Behr and Ryu 2008). Therefore, we suspect that pulmonary vascular bed alterations may be involved in PE and lower extremity vein thrombosis epidemiology. We hypothesized that patients with chronic lung diseases involving pulmonary vascular beds would have a higher prevalence of PE rather than DVT. To test the hypothesis, we investigated the prevalence of PE and DVT with chronic lung diseases and connective tissue disorders (CTDs) and within the general population.

## Methods

### Study subjects

This is a retrospective cohort study based on data collected from the Korean Health Insurance Review and Assessment Service (HIRA) national database. HIRA is a government-affiliated organization that builds an accurate claims review and is a quality assessment system for the National Health Insurance (NHI). The NHI is the only public medical insurance system operated by the Ministry of Health and Welfare in Korea (Kim 2010a, b). We evaluated the prevalence of lung cancer associated with ILD and idiopathic pulmonary fibrosis (IPF) utilizing HIRA data from January 2011 to December 2011. The database (HIRA-NPS-2011-0001) was based on a stratified random sampling of outpatients from the whole population. However, data included both outpatients and inpatients health services utilization. However, data include both outpatients and inpatients health services utilization. The database (HIRA-NPS-2011-0001) was built with first medical claims of the patients during 2011 and followed up until December 2011. Extracting patients by stratification system were based on gender, age range, as well as diagnostic and prescription history. There were 1,375,842 sample cases and 670,258 of these (age ≥40) cases were evaluated. Patients with CTD, ILD, or COPD were identified based on the International Classification of Disease-10 (ICD-10) diagnostic codes. This study was approved by the institutional review board at Dongsan Hospital, Keimyung University School of Medicine. Consent was not obtained since the patient information was anonymized.

### Case identification

Cases were included in the study based upon diagnostic and treatment evidence of ILDs and IPF. Each case was included both the diagnostic and procedure codes were identified simultaneously. Diagnosis interstitial lung disease was based on doctor’s clinical judgment based on chest radiographic findings. These processes were performed automatically using a computer-based program. The ICD-10 diagnostic codes are used as a reference for the medical diagnosis of diseases and within the health insurance system. We used the J44 (ICD-10 code) for the obstructive pulmonary disease. The diagnosis of COPD was based on clinical findings and spirometry. The codes used for the identification of systemic CTDs were as follows: M05 for rheumatoid arthritis; M07 for psoriatic and enteropathic arthropathies; M30 for polyarteritis; M31 for other necrotizing vasculopathies; M32 for systemic lupus erythematosus; M33 for dermatopolymyositis; M34 for systemic sclerosis; M35 for other systemic involvement of connective tissue; and M45 for ankylosing spondylitis.

The codes used for DVT included I80.2 (DVT, not otherwise specified [NOS]) and I80.3 (embolism or thrombosis of a lower extremity, NOS). The codes used for PE included I26 (pulmonary thromboembolism), I26.0 (PE with mention of acute cor pulmonale), and I26.9 (PE, NOS). The codes used for intra-abdominal thrombosis (IAT) included I81 (portal vein thrombosis), I82 (other venous embolism or thrombosis), I82.0 (Budd–Chiari syndrome), I82.2 (embolism or thrombosis of the vena cava), and I82.3 (embolism or thrombosis of the renal vein). The codes used for thrombosis, no specified site (TNSS) included I82.8 (embolism and thrombosis of other specified veins) and I82.9 [embolism of the vein, NOS, and thrombosis (vein), NOS]. Cases that were recorded as both DVT and PE we regarded as cases of PE. Venous thromboembolism was validated by simultaneous anticoagulation at the time of diagnosis.

### Analysis

We counted the number of patients diagnosed with ILD, CTD, or COPD in 2011 and calculated the prevalence rate. Ninety-five percent confidence intervals (CIs) were calculated using the normal approximation to the binomial distribution. A χ^2^ test was used to compare frequencies. P values <0.05 were considered statistically significant. We compared the lung cancer prevalence rates among the groups. All analyses were conducted using SAS version 9 (SAS Institute, Cary, NC, USA), and likelihood ratio tests were used for all tests of significance.

## Results

### Patients with ILDs, COPD, and controls

A total of 66 % patients with ILD and 56.3 % of COPD controls were men. The median ages of the patients with ILD and those of the COPD controls were 68 and 69 years, respectively. Approximately 2.8 % of patients with ILD and 2.3 % of COPD controls had a previous history of thromboembolism (Table 1).

**Table 1:** Demographic and clinical characteristics of patients with connective tissue disorders, chronic obstructive pulmonary disease, interstitial lung diseases, and the general population

Characteristic	General population (n = 640,177), n (%)	ILD (n = 859), n (%)	COPD (n = 15,686), n (%)	CTD (n = 7280), n (%)
Sex				
Male	299,599 (46.80)	570 (66.36)	8832 (56.30)	2105 (28.91)
Female	340,578 (53.20)	289 (33.64)	6854 (43.70)	5175 (71.09)
Age				
Median (Q1, Q3)	53 (46, 63)	68 (59, 76)	69 (60, 76)	57 (49, 68)
40–49	233,823 (36.52)	67 (7.80)	1195 (7.62)	1825 (25.07)
50–59	195,737 (30.58)	161 (18.74)	2525 (16.10)	2235 (30.70)
60–69	116,203 (18.15)	242 (28.17)	4251 (27.10)	1716 (23.57)
70–79	70,120 (10.95)	276 (32.13)	5223 (33.30)	1193 (16.39)
≥80	24,294 (3.79)	113 (13.15)	2492 (15.89)	311 (4.27)
Respiratory disease (prevalence)				
Influenza and Pneumonia (J09–J14)	3665 (0.57)	18 (2.10)	221 (1.41)	87 (1.20)
Bacterial Pneumonia (J15, J16)	6187 (0.97)	107 (12.46)	1183 (7.54)	115 (1.58)
Active tuberculosis (A15)	1121 (0.18)	25 (2.91)	335 (2.14)	30 (0.41)
Co-morbidity (prevalence)				
Malignancy (C00–C97)	32,140 (5.02)	180 (20.95)	2623 (16.72)	529 (7.27)
Diabetes (E10–E14)	108,639 (16.97)	309 (35.97)	5385 (34.33)	1894 (26.02)
Chronic renal failure (N17–N19)	7662 (1.20)	50 (5.82)	723 (4.61)	191 (2.62)
Previous myocardial infarction (I21, I22, I23, 25.2)	5983 (0.93)	35 (4.07)	578 (3.68)	114 (1.57)
Congestive heart failure (I50)	9584 (1.50)	92 (10.71)	1551 (9.89)	186 (2.55)
Varicose veins of lower extremities (I83, O22.0, O87.8)	3655 (0.57)	5 (0.58)	105 (0.67)	68 (0.93)
Hospital admission	98,780 (15.43)	482 (56.11)	7100 (45.26)	1925 (26.44)
Surgery	207,567 (32.42)	401 (46.68)	7141 (45.52)	2667 (36.63)
Atrial fibrillation (I48)	6340 (0.99)	46 (5.36)	829 (5.28)	108 (1.48)
Inflammatory bowel disease (N50–N52)	294 (0.05)	1 (0.12)	10 (0.06)	5 (0.07)

Between January 2011 and December 2011. Summarized based on the diagnosis at the top of the list from the International Statistical Classification of Diseases and Related Health Problems, 10th edition (ICD-10) codes of respiratory diseases

*ILD* interstitial lung disease, *IPF* idiopathic pulmonary fibrosis, *COPD* chronic obstructive pulmonary disease, *CTD* connective tissue disorder

### Venous thromboembolism prevalence in the groups

From the random samples obtained from the NHI database during 2011, there were 670,258 cases aged ≥40 years from a total of 1,375,842. The number of CTD and COPD cases accounted for 1.14 and 2.37 % of the sample population, respectively (Table 2).

**Table 2:** Gender distribution of the study population for deep vein thrombosis, and pulmonary embolism prevalence in the Korean sample population

Group	Population	Cases	Prevalence	95 % CI	Population	Cases	Prevalence	95 % CI
	Men				Women			
Prevalence of deep vein thrombosis (1/100,000)								
General population	299,599	418	139.5	132.7–146.3	340,578	467	137.1	130.7–143.4
COPD	8840	63	712.6	623.7–802.8	6846	37	540.6	451.3–628.3
CTD	2009	15	746.6	529.2–895.9	5271	26	493.2	404.1–600.7
ILD	571	2	350.2	103.2–598.5	288	3	1041.6	441.8–1634.2
Prevalence of pulmonary embolism (1/100,000)								
General population	299,599	319	106.4	100.5–112.4	340,578	406	119.2	113.3–125.1
COPD	8840	110	1244.3	1127.4–1363.4	6846	76	1110.1	982.3–1235.3
CTD	2009	9	447.9	285.3–569.7	5271	21	398.4	317.4–494.1
ILD	571	12	2101.5	1503.9–2706.5	288	3	1041.6	441.8–1634.2

Between January 2011 and December 2011. Summarized based on the diagnosis at the top of the list of the International Statistical Classification of Diseases and Related Health Problems, 10th edition (ICD-10) codes of respiratory diseases

*ILD* interstitial lung disease, *COPD* chronic obstructive pulmonary disease, *CTD* connective tissue disorder

Deep veins thrombosis (DVT) was detected in 885 (0.29 %) cases among those aged ≥40 years. The DVT prevalence was 135.3 per 100,000 individuals within the general population. The DVT prevalence in men with CTD was 746.6 per 100,000 individuals, which is approximately 5.3 times higher than the prevalence seen within the general population. In addition to CTD, the DVT prevalence in men with COPD and ILD was about 5 and 3 times, respectively, higher than the prevalence seen in men within the general population. However, the PE prevalence was 11–15 times higher in patients with COPD and ILD compared to the general population. The PE prevalence for patients with CTD was 1/3–1/4 of that seen for COPD or ILD patients, while the prevalence of DVT was similar among patients with CTD, ILD, and COPD.

### Deep vein thrombosis and pulmonary embolism prevalence among the groups

The odds ratios for DVT prevalence were similar among COPD, CTD, and ILD patients. However, the PE prevalence in patients with COPD or ILD was significantly higher than in patients with CTD (P < 0.01) (Table 3).

**Table 3:** Risk of deep vein thrombosis and pulmonary embolism prevalence in patients with chronic obstructive pulmonary disease and connective tissue disorder

Group	Population	Cases	Prevalence	95 % CI	Odds ratio	95 % CI
Deep vein thrombosis						
General population	640,177	885	138.2	133.6–142.8	Ref	
COPD	15,686	100	637.5	573.9–701.0	4.8	3.9–5.9
CTD	7280	41	563.1	475.4–650.8	4.0	2.9–5.5
ILD	859	5	582.0	322.5–841.6	4.41	1.8–10.6
Pulmonary embolism						
General population	640,177	725	113.2	109.0–117.4	Ref	
COPD	15,686	186	1185.7	1009.3–1272.2	11.3	9.6–13.2
CTD	7280	30	412.0	337.0–487.1	3.61	2.5–5.2
ILD	859	15	1746.2	1299.3–2193.1	16.39	9.7–27.4

*PE* pulmonary embolism, *CTD* connective tissue disorder, *COPD* chronic obstructive pulmonary disease

### Pulmonary embolism-to-deep vein thrombosis prevalence ratio among the groups

**Fig. 1:**
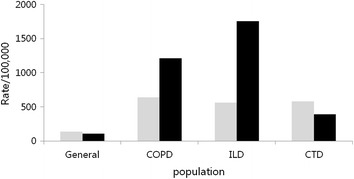
Deep vein thrombosis (DVT) and pulmonary embolism (PE) prevalence rates between the groups. *COPD* chronic obstructive pulmonary disease, *ILD* interstitial lung disease, *CTD* connective tissue disorder. *Gray bar* denotes DVT and *black bar* denotes PE

The PE-to-DVT prevalence ratio in the general population was 0.78. The ratios for COPD, ILD, and CTE were 1.8, 3.1, and 0.66, respectively. The prevalence of PE was the highest in patients with ILD (Fig. 1).

## Discussion

This is a population-based study, which provides new data supporting the association of chronic lung diseases such as ILD and COPD with PE. The PE prevalence rates per 100,000 individuals from the study population with COPD, ILD, CTD without ILD, and the general population were 1185, 1746, 412, and 113, respectively, while the DVT prevalence rates were 637, 582, 563, and 138, respectively. The prevalence rates of VTE in patients with COPD, ILD, CTD without ILD, and the general population were 2339, 2793, 1346, and 482 per 100,000 individuals.

CTDs have been associated with an increased risk of PE and DVT development (Choi et al. 2013; Zoller et al. 2012; Ramagopalan et al. 2011; Matta et al. 2009). The risk of DVT in CTD patients was approximately 2–3 times higher than within the non-connective tissue disorder population (Choi et al. 2013; Zoller et al. 2012; Ramagopalan et al. 2011; Matta et al. 2009). In the present study, we evaluated all of the CTDs in the sample population and discovered that the odds ratio for developing DVT in CTD patients is approximately 3.6–4.0 compared to the general population. The risk of developing DVT in CTD patients found in our study is slightly higher than that found by previous studies, which is partly influenced by selection bias from the sample population. However, the results of present study are comparable to a previous study (Ramagopalan et al. 2011).

ILDs have been associated with an increased risk of VTE development (Sprunger et al. 2012; Hubbard et al. 2008; Sode et al. 2010). The risk of DVT in ILD was approximately 2–3 times higher than that found within the control population (Hubbard et al. 2008). The PE prevalence was higher in COPD patients (Tillie-Leblond et al. 2006; Rizkallah et al. 2009). In the present study, the risk of developing DVT or VTE in ILD and COPD patients was 4–6 times higher than that found within the control population.

The incidence of PE and DVT was similar in rheumatoid arthritis patients (Choi et al. 2013). However, the prevalence of PE was approximately 2 times higher than that of DVT in the COPD population (Tillie-Leblond et al. 2006; Rutschmann et al. 2007). In the present study, the prevalence of DVT was higher than PE in the general population and in CTD patients. In contrast to the general population and CTD patients, PE prevalence was 2–3 times higher than DVT in COPD and ILD patients. There are two possibilities as to why there was a higher prevalence of PE than DVT in ILD and COPD patients. First, COPD and ILD patients have thrombogenic potential within the pulmonary circulation (Magee et al. 1988; Wright et al. 1992; Keane et al. 1997; Cosgrove et al. 2004; Renzoni et al. 2003; Sakao et al. 2006). Therefore, COPD and ILD patients have a chance to develop in situ thrombosis. Another possibility is that vascular bed changes may cause delayed resolution of or no resolution of PE from DVT. The pulmonary artery pressure is 2–3 times higher than the central venous pressure, which means that blood flow is faster in the pulmonary artery than in the peripheral vein. The pulmonary artery, from the pulmonary circulation, accounted for 46 % of the total pulmonary resistance (Brody et al. 1968). From this point of view, stagnation of blood in a lower extremity is different from that experienced by the pulmonary artery. The arterial blood coagulation factor has a shorter length of stay in comparison to the venous coagulation factor, resulting in a relatively lower level of coagulation factors found in the artery. The exposure intensity of blood coagulation factors in the pulmonary vein is assumed to be low in comparison to that found in the lower extremity vein. However, this physiologic phenomenon may be reversed by lung local factors (Keane et al. 1997; Cosgrove et al. 2004; Renzoni et al. 2003; Sakao et al. 2006; Chaouat et al. 2008; Behr and Ryu 2008). This may explain the discrepancy of venous thrombosis prevalence between the lower extremity and pulmonary artery with or without the presence of chronic lung diseases.

The association between PE and infection is supported by several studies (Levi et al. 2010; Levi 2004; D’Angelo et al. 1988). Inflammatory condition may contribute the development of venous thrombosis. PE and DVT was significantly associated with COPD exacerbation as well as mortality (Gunen et al. 2010). The odds ratio of PE ranged from 1.7 to 5.0 in inflammatory conditions (D’Angelo et al. 1988; Bucciarelli et al. 1999; Wells et al. 2005). An inflammatory condition in conjunction with structural abnormalities may explain the high prevalence of pulmonary embolism in COPD and ILD.

In 2008, population-based statistics demonstrated that the PE and DVT incidence in the whole population was 5.3 and 7.0 per 100,000 individuals, respectively (Jang et al. 2011). When we confine this to those older than 40 years of age, DVT and PE reaches 13 and 24 per 100,000 individuals, respectively. The incidence of PE based on the number of hospitalized adults was 100–200 fold higher than that found in a population-based study in Korea, which is similar to the present study’s findings of PE prevalence (Choi et al. 2012). This population-based study used the whole population as the denominator. However, this HIRA data was based on individuals who visited a clinic at least once in the 2011 calendar year. This may lead to an overestimation of lung cancer prevalence in the HIRA data or an underestimation of venous thrombosis in the national statistics. Although there would be a discrepancy between this sample population data and the national data, a comparison of the PE prevalence among the groups in the sample data would be possible.

The man-to-woman VTE cancer ratio was 1.0 in those within the background population. The ratios of COPD, ILD, and CTD were 1.1, 1.2, and 1.0, respectively. There was no significant difference between the groups. We suspect that the prevalence of VTE is less influenced by sex even in patients with CTDs.

The HIRA database provided limited available patient data regarding age, sex, year, diagnostic code, and medication code. Therefore, we could not accurately validate patients by identification or exclusion of definition through review of the source medical records. The severity of patients with venous thromboembolism was not evaluated due to the limitation of access to the individual medical records. However, this large-scale data may provide new insights of PE prevalence in patients with COPD and ILD.

In conclusion, our study suggests that the PE prevalence in relation to DVT was significantly higher in patients with COPD or ILD than in patients with CTD without ILD or within the general population. Furthermore, our findings suggest that local inflammatory and physiologic factors may contribute to the development of PE.
